# Models with environmental drivers offer a plausible mechanism for the rapid spread of infectious disease outbreaks in marine organisms

**DOI:** 10.1038/s41598-020-62118-4

**Published:** 2020-04-06

**Authors:** E. A. Aalto, K. D. Lafferty, S. H. Sokolow, R. E. Grewelle, T. Ben-Horin, C. A. Boch, P. T. Raimondi, S. J. Bograd, E. L. Hazen, M. G. Jacox, F. Micheli, G. A. De Leo

**Affiliations:** 1 Hopkins Marine Station, Stanford University, Pacific Grove, CA USA; 2 U.S. Geological Survey, Western Ecological Research Center, at Marine Science Institute, University of California, Santa Barbara, CA USA; 3 Haskins Shellfish Research Laboratory, Rutgers University, Port Norris, NJ USA; 4 Monterey Bay Aquarium Research Institute, Moss Landing, CA USA; 5 University of California, Santa Cruz, CA USA; 6 NOAA Southwest Fisheries Science Center, Monterey, CA USA; 7 Stanford Center for Ocean Solutions, Pacific Grove, CA USA

**Keywords:** Ecological epidemiology, Ecological modelling

## Abstract

The first signs of sea star wasting disease (SSWD) epidemic occurred in just few months in 2013 along the entire North American Pacific coast. Disease dynamics did not manifest as the typical travelling wave of reaction-diffusion epidemiological model, suggesting that other environmental factors might have played some role. To help explore how external factors might trigger disease, we built a coupled oceanographic-epidemiological model and contrasted three hypotheses on the influence of temperature on disease transmission and pathogenicity. Models that linked mortality to sea surface temperature gave patterns more consistent with observed data on sea star wasting disease, which suggests that environmental stress could explain why some marine diseases seem to spread so fast and have region-wide impacts on host populations.

## Introduction

In summer 2013, sea stars off the Washington coast began “falling off the rocks – dead by the thousands”^1^. When similar reports came in from Alaska to Baja California, it became clear this was not an isolated event. Other large-scale marine disease outbreaks include white plague affecting reef-building corals across the Caribbean and Western Atlantic^2,3^, black abalone mass mortalities throughout southern and central California^4^, and the infamous Caribbean sea urchin die-off of the 1980s^5^. Several hypotheses could explain how sea star wasting disease (SSWD) and other marine disease outbreaks occur so rapidly over such vast distances. For instance, some marine pathogens can spread faster than terrestrial pathogens via water currents or long-distance host movement^6^. However, juvenile and adult sea stars move slowly, and the virus associated with SSWD was potentially detected using NS1/VP4 qPCR in sea star tissue samples dating back to 1942, although its past presence was not associated with mass mortality and large-scale disease outbreaks^7,8^. The infectious agent might not be new, but the continental-scale die-offs appear to be.

Marine disease outbreaks are often hypothesized to increase under environmental stress^9^. On the other hand, infectious agents have their own thermal limits, and increased temperature can thereby reduce disease under some scenarios^10^. Large-scale, basin-wide events, such as the El Niño Southern Oscillation (ENSO) and the recent warm-water anomaly of 2013-16^11–13^, can lead to rapid changes in temperature^14^, salinity^14^, pH^15^, nutrients^16^, and oxygen^17,18^ that can stress marine organisms. Stress might lower host defenses, speed disease development, and expand pathogen home ranges^14^. For example, warm temperatures and high salinity increase mortality in *Perkinsus marinus* (Dermo) and *Haplosporidium nelsoni* (MSX) infected oysters; along the Atlantic coast, warmer winter temperatures have expanded these parasites northward^19^, and higher mortality occurs during the positive phase of the North Atlantic Oscillation^20,21^. In the warmer Gulf of Mexico waters, salinity fluctuations associated with ENSO govern disease expression^21^. Similarly, although withering syndrome die-offs in Pacific coast abalone occur during both warm and cool years, pathogen-induced mortality rates are higher during warmer years^4^.

In the last decade there has been an increasing recognition of the influence of day-to-day climate variability on the dynamics of infectious diseases^22–24^. The effects of temperature, humidity, and precipitation on disease incidence have been documented for waterborne diseases (e.g., cholera^25–27^) and vector-borne diseases (e.g., malaria and dengue virus^28–32^), especially for human pathogens with an important environmental component in their transmission cycle. Similar climate effects have been observed in wildlife diseases both terrestrial^33–35^ and marine systems^14^. The effects of climate on disease transmission cannot be easily isolated through statistical analysis, however, because the size of disease outbreaks may depend on both climate influences and the abundance of susceptible and infectious individuals in the host population, which interact in a non-linear fashion to produce the observed disease dynamics^26^. In addition, as the climate-disease relationship may vary between different regions, it is critical to investigate the environmental drivers of diseases by integrating spatial and temporal dynamics in a single modelling framework. With a few notable exceptions^27,36^, the majority of studies of human infectious disease have either spatially aggregated data when analyzing variance of incidence over time^25,26^ or analyzed regional-scale variations in prevalence purely as a function of climate drivers without accounting for temporal dynamics in the abundance of susceptible and infected populations. Additional complexities might arise when dispersal of infectious stages is influenced, as in our case, by spatial-temporal heterogeneities in other environmental drivers such as ocean currents. Therefore, to overcome some of the limitations of regression analysis of incidence data as a function of temperature, it is crucial to explore the potential interactions of climate and disease with spatial-temporal dynamical models.

## Sea Star Wasting Disease (SSWD) as a model system

**Figure 1:**
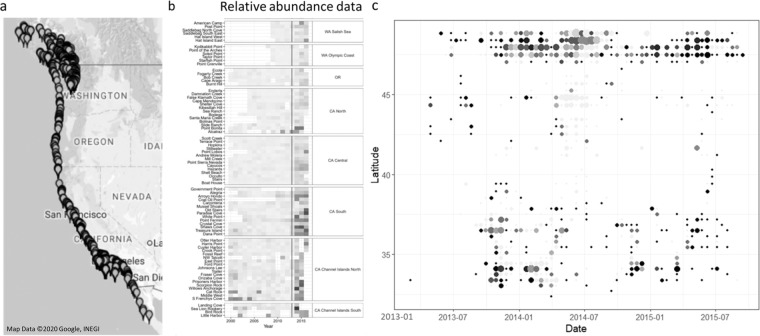
Spatial outbreak pattern for sea star wasting disease in the ochre seastar *Pisaster ochraceus*. All observational data were gathered via a Citizen Science sampling initiative, in combination with the Multi-Agency Rocky Intertidal Network (MARINe), and are available on www.seastarwasting.org. Observations were in the form of species-specific *presence/absence* data, with sampling from both long-term research sites and citizen-selected locations. (**a**) Survey locations along the Pacific coastline of North America^66^. (**b**) Sea star density relative to pre-SSWD at multiple survey sites, with darker colors indicating greater decline (figure from Miner *et al*. 2018). The vertical line indicates the start of the SSWD epidemic. (**c**) The aggregation of survey data into 50 km × 14 day “windows”. The x-axis indicates time in days, with day 1 corresponding to January 1^st^, 2013, and the y-axis indicates latitude of 5 km cells, the resolution of the simulation. Proportion of “presence” surveys in each window is indicated by color, with brighter points showing a higher proportion.

In just four months, SSWD appeared at scattered sites from Alaska to the Mexican border, then filled in the gaps (Fig. 1a). Hewson *et al*.^7^ proposed that SSWD might be caused by a virus associated with dying sea stars (though the definitive agent remains in doubt^8^). But the distance between consecutive outbreaks varied over time, and was often far, raising speculation about how a virus could move such distances so rapidly. Although no clear environmental trigger has been identified, temperature correlates with infected mortality rates in adult sea stars^37,38^, and some die-offs occurred during when it was warmer than usual (e.g., in the Channel Islands^39^ and Washington^40^ though not Oregon^41^). Some evidence suggests that stress could have contributed to heightened mortality^8,42^. In particular, population decline was much greater along the warmer southern California coast than to the North (Fig. 1b)^43^. However, mass mortalities of *Pycnopodia helianthoides* at cold sites suggest that stress is more likely associated with a temperature anomaly than absolute temperature, per se^42^. Overall, the correlation between SSWD and high temperature is complicated and inconsistent, with no clear link to disease emergence. As a complement to these studies, we contrasted possible mechanistic models for widespread marine epidemics like SSWD with different degrees of temperature-dependence.

This study is an unprecedented analysis of presence-absence data on SSWD, using fine-scale spatial and temporal resolution, that we have used to investigate alternative hypotheses on the effect of temperature anomalies and oceanographic drivers on disease dynamics. Specifically, we have contrasted a coupled oceanographic-epidemiological model in which infection process and pathogenicity are described through constant rates and dispersal is driven only by ocean currents with two alternative models in which (a) transmission rate – the rate at which susceptible becomes infected – is a function of temperature and (b) pathogenicity – the rate at which an asymptomatic infection develops into a full fledge-infection – is a function of accumulated thermal stress.

Our core oceanographic-epidemiological model is spatially-explicit with a susceptible class, an infected but asymptomatic class (exposed), a symptomatic (infective) class exhibiting high mortality rates, dispersal of infectious propagules driven by ocean currents and a range of assumptions about the influence of temperature on the rate at which new infections were recruited in the exposed class and how they transitioned from asymptomatic (exposed) to symptomatic, full-fledged infections. Specifically, we first analyzed the spatial-temporal dynamics of a temperature-independent epidemiological model in which diffusion/advection of infective propagules was driven entirely by oceanography along the North American Pacific coast. We then compared the dynamics of a second model in which, in addition to current-driven propagule dispersal, infection rate in the exposed but asymptomatic class increased linearly with temperature variance. Finally, we analyzed a third model in which transition from asymptomatic to full-fledged infection was triggered by accumulated stress following repeated or prolonged exposure to high temperatures. The two latter models were inspired by the environmentally-triggered transmission and pathology experienced by intertidal black abalone (*Haliotis cracherodii*)^44,45^ and documented in other marine disease systems, such as the oyster *Crassostrea gigas* and Ostreid herpesvirus type 1^46,47^. The aim of our work was not to simulate the SSWD outbreak in detail, but to outline what transmission mechanism is most likely to reproduce spatiotemporal patterns similar to those observed in the 2013–15 outbreak. We used a data-assimilative regional ocean model^48^ to estimate sea surface temperature and current direction and speed (coinciding with an extensive warm-water anomaly^11^) and a maximum likelihood estimator to explore support for different model assumptions.

## Materials and Methods

### Epidemiological model

**Figure 2:**
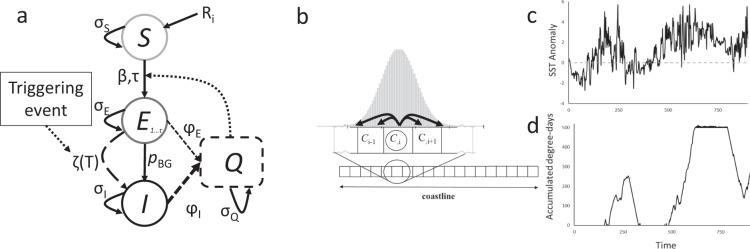
(**a**) Within-cell model outline. Susceptible individuals (*S*) have constant daily recruitment *R*_*i*_ and survival σ_S_ and become exposed (*E*) τ days after interaction with disease propagules (*Q*) at a rate β. Exposed individuals have survival σ_E_ and transition to symptomatic individuals (*I*) at a constant rate *p*_BG_ and/or as a function of accumulated degree days, ζ(*T*). Symptomatic individuals have low survival (σ_I_ ≪ σ_E_) and produce propagules at a higher rate than exposed individuals (*φ*_I_ > *φ*_A_). Disease propagules have a daily persistence of σ_Q_ that is not noticeably diminished by infection. (**b**) Dispersal of disease propagules along a linear, uniform coastline. The dispersal kernel is normal and symmetric, with absorbing boundaries at the ends of the coastline. For any specific cell and day, the mean distance of the normal kernel is determined by mean along-shore current from the ROMS model. (**c**) An example average daily sea surface temperature anomaly time series from roughly halfway up the coastline, 2013–2015 (cell 300 of the ROMS model), with the zero level shown as a dashed line. (**d**) Associated within-cell degree-day accumulation and recovery. The triggering threshold is at the top of the y-axis.

The model simulates disease dynamics on a linearized Pacific coastline divided into 500 5-km cells, each representing a local sea star population subject to infective propagules (pathogens) that disperse, through diffusion/advection, between neighboring cells according to local ocean currents (Fig. 1c; linearization described in Appendix A). Following the consumer-resource modelling structure presented by Lafferty *et al*.^49^, the sea star population in each cell was described by a compartmental model with three infection classes – susceptible, *S*, recruited at a constant daily rate; exposed but asymptomatic, *E*; and infected and symptomatic, *I*. Cell carrying capacity and recruitment varied by region (Table C2). The compartment *Q* represents free-living infective stages or propagules dispersing through water in nearby spatial blocks (i.e., cells), which altogether form a Q-SEI modelling framework^49^. As pathogen propagules reached a cell, a fraction of susceptible hosts became infected but were initially asymptomatic, producing pathogen propagules at a low background rate that dispersed through a combined diffusion/advection process (*sensu* Skellam^50^; Fig. 2a,b). In contrast, once symptomatic, individuals were assumed to die in just few days (thus exhibiting high mortality rate) and have a high disease propagule production, similar to observations in other marine disease systems such as Dermo in oysters^51^.

The infection rate – i.e., the rate at which new, initially asymptomatic infections are recruited following exposure to infective propagules – was assumed to be either constant or an increasing function of temperature variance, similar to the response documented for withering syndrome in abalone^44^. Likewise, the daily transition to a diseased state (i.e., from the *E* to the *I* class) was assumed to either be a constant, temperature-independent rate or to occur when accumulated stress, generated by repeated exposure to temperature anomalies, exceeded a given threshold. In the latter case, the underlying hypothesis was that thermal stress reduces the host’s ability to control a pathogen by compromising its immune response, that the pathogen replication rate increases with temperature faster than the host’s immune response, or a combination of both^52^. It follows that, at higher-than-normal temperatures, the pathogen is eventually able to take over its host, a phenomenon referred to in the literature as thermal mismatch hypothesis^53,54^. Here, we used the accumulated degree-days above a stress threshold as proxy-indicator of thermal stress for an infected but asymptomatic sea star (Fig. 2c,d). We combined these mechanisms to create three classes of models: *Const*-Q-SEI is a classic transmission model with no temperature dependency and dispersal of infective propagules driven only by ocean currents; *EnvInf*-Q-SEI is similar to *Const*-Q-SEI but with high temperature variation increasing the infection rate, which regulates the transition from susceptible to the infected & asymptomatic class (S to E) following exposure to infective propagules; and *EnvDr*-Q-SEI, an environmentally-driven model similar to *EnvInf*-Q-SEI but with, in addition, the transition from asymptomatic to full-fledged infection (E to I) driven by accumulated temperature stress. Appendices B and C describe the model and parameter values, and Appendix D reports sensitivity analyses that show that the qualitative spatial-temporal patterns generated by each of these models are not sensitive to the specific values of model parameters.

In the *Const* and *EnvInf* models, we simulated disease dynamics following the introduction of an infected individual in an otherwise completely naïve population. The first infection was assumed to occur approximately where the first case of SSWD was reported in the United States in 2013, namely Half Moon Bay, California. In the *EnvDr* model, the pathogen was assumed to have already spread along the coastline infecting the entire population asymptomatically prior to the first appearance of the disease (following the hypothesis that the pathogen was widespread and not novel). We then simulated disease dynamics in time and space following site-specific accumulation of thermal stress, starting in 2013 in order to directly compare with the other two models. Because of data limitations and computational complexity, we do not consider in this manuscript the questions of whether disease could have been triggered in prior years or how incomplete pathogen spread and disease expression could interact. Daily temperature and infective propagule dispersal along the coastline were simulated using mean sea surface temperature and along-shore surface velocity from a data-assimilative implementation of the regional ocean modeling system (ROMS) configured for the California Current System (oceanmodeling.ucsc.edu/ccsnrt)^48^. In all cases, disease dynamics were simulated for 1000 days between January 2013 and December 2015.

We hypothesized that accumulated stress from repeated exposure to high temperature anomalies (defined as positive deviations from local mean seasonal cycle relative to 1999–2011 climatology) can cause stronger impacts than exposure to an isolated high temperature event^55^. Consequently, we modeled accumulated thermal stress by summing the daily number of degrees of high temperature anomaly raised to an exponential power. We based this formulation on the assumption that stress has a non-linear response to temperature, as observed, for example, for oyster mortality^56^ and mussel growth^57^ and respiration rates^58^, and in accordance with empirically-derived thermal performance curves showing that physiological performance sharply decreases (and, thus, stress sharply increases) when temperature exceeds the thermal optimum^59,60^. In Appendix D, we show that the qualitative spatial-temporal pattern generated by the temperature-sensitive models was not highly sensitive to the precise form of the stress accumulation curve, provided that stress due to temperature anomalies accumulates over time. When the accumulated degree-days at a cell location exceeded a triggering threshold, asymptomatic *E* individuals transitioned into the symptomatic class, *I* (Fig. 2d). During periods of lower temperature, accumulated stress decreased as the population in that cell recovered. Although outbreaks are smaller and more frequent for smaller threshold values, the qualitative spatial-temporal dynamics did not depend upon the specific value of this threshold (Appendix D).

### Analysis

We assessed our hypotheses by comparing a model’s output with observed data for the SSWD outbreak, focusing on two principal metrics for an epidemic: prevalence of the disease, and relative decline in individuals. For disease prevalence we used SSWD *presence/absence* data from MARINe surveys collected 2013–2015 (Fig. 1c; www.seastarwasting.org), and for relative decline we used sea star abundance data from Miner *et al*.^43^ (Fig. 1b). We assessed the match between model and data across time and space using a *maximum likelihood estimator* (MLE), for which a lower value of the log likelihood estimator indicates a greater likelihood that the processes described by a specific model produced the epidemiological data that were actually observed between 2013–2015. For the *presence/absence* data, we divided both the data and model output into 14 day × 50 km time-space “windows” to capture each section of the linearized coastline as it changed through time, then calculated and summed the MLE values for all regions across the 1000 days of the simulation (see Appendix E for MLE calculation details). The match with levels of sea star abundance was calculated similarly, except that we standardized the data to be decline relative to 2013 abundance and were limited to comparing 2014 and 2015 with 2013 because of its coarse resolution. The fit of each model as thus assessed independently against both epidemiological metrics.

Because the MARINe surveys were not evenly distributed in space and time, we created an additional *presence/absence* MLE value to check the robustness of our results after taking the uneven sampling into account. To do this, we stochastically resampled model output to match the number of observations in the survey data (*n* = 1862) and their spatial and temporal distribution, simulating an equivalent distribution of *presence/absence* data, and used the simulated survey data to assess MLE values. We repeated this resampling 100 times to estimate the standard error for each metric.

## Results

### Potential effects of environmental triggers on disease dynamics

**Figure 3:**
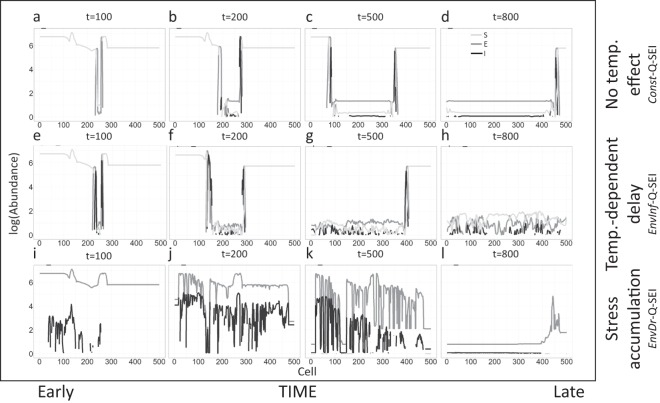
Disease spatial spread under the *Const*-Q-SEI (**a**–**d**), *EnvInf*-Q-SEI (**e**–**h**), and *EnvDr-*Q*-*SEI (**i**–**l**) models. (**a**) Abundance of individuals at t = 50 days under the standard *Const*-Q-SEI model. The x-axis indicates position along the coastline and y-axis shows log-abundance (base 10) of S (light gray lines), E (dark gray lines), and I (black lines) individuals. (**b**) Abundance at t = 100. (**c**) Abundance at t = 200. (**d**) Abundance at t = 400. (**e**–**h**) Same as (**a**–**d**), except under the *EnvInf-*Q*-*SEI model. (**i**–**l**) Same as (**a**–**d**), except under the *EnvDr-*Q*-*SEI model.

The *Const*-Q-SEI model with no temperature dependency and dispersal of infective propagules driven only by ocean currents generated outbreaks that spread across the landscape as a traveling wave (dark gray and black lines, Fig. 3a–d). Disease spread faster to the south (lower-numbered cells) than the north, reflecting that along-shore currents headed predominantly to the south during the study period. The high disease-induced mortality prevented the hypothetical host population from recovering to its pre-epidemic abundance, yet daily recruitment provided new susceptible individuals. Therefore, the system reached a stable and spatially uniform endemic equilibrium along the coastline (Fig. 3d), with the fraction of *S*, *E* and *I* individuals equal to 8.7%, 89.9% and 5.4% respectively, and population abundance remaining <0.001% of disease-free carrying capacity.

The *EnvInf-*Q*-*SEI model (temperature-dependent infection rate) generated dynamics similar to the *Const*-Q-SEI model, but the epidemic front moved more rapidly, particularly to the south (Fig. 3e–h) where warmer temperatures increased transmission rate and accelerated the transition from *S* to *E*. Under this scenario, the sea star population reached a low-abundance equilibrium state with frequent fluctuations driven by a combination of non-linear transmission rates and the influence of temperature on disease transmission (Fig. 3h).

In contrast, the *EnvDr*-Q-SEI model (*E*-to-*I* transition triggered by accumulated temperature stress) was initialized with the pathogen having historically established asymptomatically along the entire coastline (dark grey line, Fig. 3i), with only a few scattered early signs of disease (black line). As the summer progressed, local disease outbreaks popped up after accumulated stress in a given location (i.e., in a cell of the discretized spatial domain) exceeded the threshold, triggering a quick transition from asymptomatic (*E*) to symptomatic (*I*) class and full-fledged infection (Fig. 3j). As time progressed and more extreme-temperature degree-days were again accumulated in cells across the coastline, outbreaks were almost synchronous in time across vast distances, but with little discernible spatial pattern (Fig. 3k). Eventually, at the end of the simulation time, the disease had wiped out almost the entire population except a few cooler-water populations in the north (Fig. 3l).

### Comparison with observed SSWD dynamics

**Figure 4:**
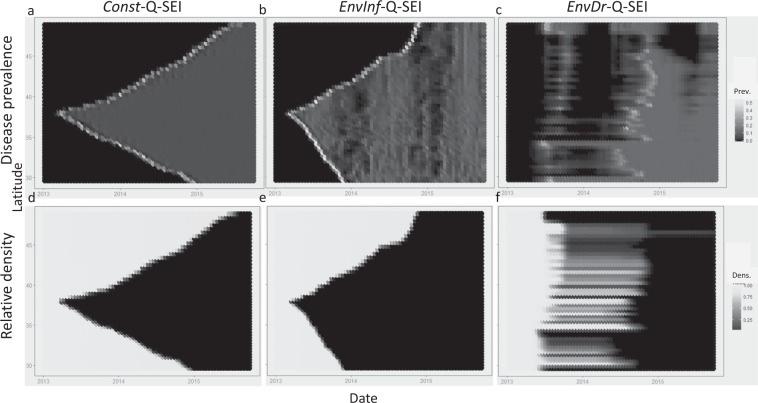
Simulated spatial dynamics generated by the *Const*-Q-SEI, *EnvInf*-Q-SEI, and *EnvDr*-Q-SEI models. In the first two scenarios, the first infected individual is introduced into cell 250 at t = 20, the middle of a coastline discretized into an arbitrary *n* = 500 cells. For the *EnvDr-*Q-SEI model, the infection is pre-existing along the coastline. (**a**) Disease prevalence map for *Const*-Q-SEI model. The horizontal axis indicates time in days, the vertical one latitudinal location along the coastline. Shading indicates proportional prevalence of the symptomatic *I* class with darker colors representing lower prevalence. (**b**) Same as 4a, except for the *EnvInf*-Q-SEI model. (**c**) Same as 4a, except for the *EnvDr*-Q-SEI model. (**d**) Relative density map for the *Const*-Q-SEI model. Shading indicates abundance of all disease classes relative to initial pre-SSWD density, with dark colors representing low relative density. (**e**) Same as 4d, except for the *EnvInf*-Q-SEI model. (**f**) Same as 4d, except for the *EnvDr*-Q-SEI model.

To qualitatively compare model outputs with SSWD observations, we created spatial abundance maps across time (Fig. 4a–f). Disease spread in the *Const*-Q-SEI model was followed by a sharp decline in host density (Fig. 4a,d). The *EnvInf-*Q*-*SEI model showed a similar but faster spread (Fig. 4b,e). The *EnvDr-*Q-SEI model generated spikes in mortality fragmented in space and pulsed in time due to the fine-scale spatial heterogeneity in temperature stress accumulation (Fig. 4c,f).

We evaluated each model against the *presence/absence* and abundance decline data to assess maximum likelihood (Table 1). Both the decline data and prevalence data were best supported by the *EnvInf*-Q-SEI model in the baseline scenario, but the *EnvDr*-Q-SEI model had better support for the prevalence data when accounting for uneven survey distribution via stochastic resampling. The results were robust across many different values of *S*-to-*E* transition delay, disease transmission rate, initial infection location, and stress accumulation parameters (Appendix D). Although the analyses did not conclusively identify one of the two temperature-based transmission mechanisms as the most likely, both models based on environmental drivers outperformed the classic *Const-*Q*-*SEI model in explaining SSWD observations. Because of the difficulty of comparing parameter counts with models of differing structure and with values not fit to the data, we did not apply a model selection process to the MLE comparison. However, preliminary analysis with simplified Akaike Information Criterion^61^ values did not produce any change in the relative ranking seen in Table 1.

**Table 1:** Model comparison.

Metric	*Const*-Q-SEI	*EnvInf*-Q-SEI	*EnvDr*-Q-SEI
Relative decline MLE	17444	**−0.05**	586
Presence/absence MLE	4330	**3873**	4138
Resampled presence/absence MLE	4897 +/− 5	4722 +/− 9	**4292** +/− **10**

## Discussion

Although the role of temperature on the emergence of SSWD remains controversial^8,40,41,43^, our models were a better fit to presence-absence data on SSWD at a fine-scale spatial and temporal resolution if they accounted for temperature effects on transmission and pathogenicity, supporting the hypothesis that small-scale climatic variability influences SSWD spatial dynamics. We evaluated model performance using both disease prevalence and sea star abundance patterns across the coastline, allowing us to account for possible spatial correlation in disease dynamics. Here, outbreak speed and spatiotemporal synchronicity resulted from an interaction between nonlinear-infectious processes and environmental stress. Additionally, comparing simulation output of different models allowed us to assess the relative likelihood of different disease transmission mechanisms despite the high variance and limitations of the data.

An environmental trigger implies that disease-induced mortality may increase abruptly with environmental change. Two features may drive such non-linearity in our system. The first is that disease-induced mortality may increase more than linearly with temperature when a host’s thermal optimum is exceeded, reflecting the non-linear and generally left-skewed shape of thermal tolerance curves^59^. The second is the non-linear dynamic in the race between the pathogen’s exploitation of the host and the host immune response, which produces an abrupt mass die-off when the accumulated thermal stress exceeds a given threshold above which the hosts are unable to control pathogen’s proliferation and develop a full-fledged infection. Thermal stress can impair the host immune response in other diseases^23,62^, and, in ectotherm hosts, temperature might often increase pathogen replication within the host^52^. That virulence may depend upon a combination of host-specific immune response and temperature effects has been clearly documented in the case of abalone Withering Syndrome^45^. More laboratory and observation-based studies will be required to clarify the relative contribution of these mechanisms. However, differential responses to high temperatures in northern, cold-water-adapted sea star populations might explain why the first occurrence of SSWD outbreaks occurred at northern latitudes. Furthermore, other environmental stressors, such as negative temperature anomalies and anomalies in salinity or oxygen, that might impair immune response or facilitate disease transmission.

The sporadic epidemiological data for SSWD makes it challenging to distinguish between an environmental stressor that indirectly causes a die-off by triggering mortality in asymptomatic infected hosts, or a stressor that directly kills individuals (whether infected or not), such as would occur with exposure to lethal temperatures or toxic algal blooms. However, in the case of SSWD, the rapid, highly symptomatic progression of wasting disease in combination with field observations of an associated pathogen^7^ suggests that, at a minimum, a prevalent and opportunistic infectious agent was able to take advantage of the environmentally-stressed host. This problem mirrors the challenge faced by pathologists when assessing dead and dying marine animals – it is often hard to distinguish a lethal disease from opportunistic infections in already dying hosts.

Though inspired by SSWD, our model represents a simplified system with which to compare patterns expected from changes in disease transmission and pathogenicity mechanisms. As occurs with most infectious disease models, we assumed homogenous host populations with no age/size structure, an abrupt mortality response to symptomatic infection, and no potential for adaptation to the pathogen over time (though genetic shifts have been observed^63^). The model didn’t account for potential demographic mechanisms driving sea star recruitment, population density regulation and size- or age- dependent demographic processes. Deviations from these assumptions could introduce new complexities that contribute to the spatiotemporal patterns of SSWD. For example, some of the patchiness seen in the SSWD event of 2013–2015 could have been due to spatial variation in the sea star community and in the patchy distribution of sea star habitat, with long-distance connectivity not captured by mean along-shore currents. Sea star larval dispersal is broad, spatially heterogeneous on a fine geographical scale, and highly variable from year to year. There are no models to our knowledge that simulate sea star recruitment at this broader geographical scale, and thus we took the simplifying assumption that there is a background rate of recruitment at the coarse spatial scale that we discretized to the coastline. The oceanographic model we used represents the mean current within 10 km of the shore, not the immediate near-shore oceanographic patterns that may be most relevant to dispersal of intertidal propagules. Current data at that spatial and temporal resolution is not available for the California-Oregon coast. Furthermore, SSWD affected many sea star species, most of which live in the subtidal habitat that is more sparsely monitored compared to the intertidal and could constitute a possible undocumented pathogen reservoir with a potential temperature refuge.

Because there are many possible model variants, in Appendix D, we show that alternative stress-accumulation functions yield similar qualitative outbreak patterns and similar analysis metric values. We also show that conclusions are robust to whether or not thermal stress affects susceptible as well as infected hosts. A model in which disease dynamics are driven entirely by ocean currents and not by thermal stress generates outbreaks occurring in the form of travelling waves that are incompatible with both the observed spatial-temporal distribution of infected sea stars and the reduction in sea star abundance, thus suggesting that other factors, such as environmental drivers and possibly thermal stress, might have played a role in generating the observed pattern. Further studies following the approach described by Koelle *et al*.^26^ and Rohr and Raffel^34^, which use dynamical models and analysis of residuals to separate climate drivers from other temporally- and spatially-confounded variables offer great potential.

Although we parameterized sea star demography from field observations, we did not incorporate possible variance or geographical differences in these estimated values due to computational complexity. Instead, we tested model sensitivity using Latin hypercube sampling of parameter values related to recruitment, survival, propagule production and dispersal, and disease transmission (Appendix D). This sensitivity analysis found that the qualitative predictions were robust to parameter uncertainty (Fig. D2).

SSWD occurred along a substantial temperature gradient from Alaska to Baja California, suggesting that the stress trigger was not absolute temperature. Through larval retention and environmental acclimation^64^, sea star communities may be locally adapted to temperature but stressed by unusual deviations, as in our model. A temperature anomaly was established in the model with respect to 1999–2011 conditions; given the 2013 appearance of a persistent warm water “blob” in the North Pacific and a strong El Niño starting in 2014^13^, the high temperatures observed represent true deviations from typical conditions for these organisms. Regardless, absolute temperatures could also be important. For instance, black abalone mass mortalities spread more slowly to the north into colder waters than to the south^4,65^.

The observational data have some limitations. The *presence/absence* data do not track SSWD prevalence and, because sampling was not systematic and relied heavily on assessment by non-scientists, there are likely unreported outbreaks, especially at less-visited sites. Sampling effort could also increase in response to die-offs. The annual relative abundance data were systematically gathered but are more limited in count and were not coordinated with the SSWD surveys. Because of this, adding a latent-class (for which pathology progresses only after accumulated stress) improved model fit to abundance data but not disease occurrence data. Due to noise and variability in marine outbreak surveillance data, it remains difficult to pinpoint the relative contributions of contagious transmission, environmentally-triggered pathogenicity, or direct (noninfectious) stress-induced mortality – or some combination of all three mechanisms – among other drivers in these die-offs.

Our results suggest that environmentally-driven disease can explain how a pathogen can persist, disperse widely, and appear in temporally synchronous mass-mortality events separated by vast distances, without requiring fast-moving pathogens. Sea star mass mortalities have been reported in the past, though none have been documented on such a massive spatial scale or across such a broad suite of species. The question of whether SSWD signals a new threat associated with environmental change or just an expected outcome of random temperature anomalies remains open.

## Data avaliability

The data and code involved in this analysis are available at 10.25740/rq831rt1470.

## Supplementary information

Appendices A-E.
