# Supplementary material for: Models with environmental drivers offer a plausible mechanism for the rapid spread of infectious disease outbreaks in marine organisms
Source: Sci Rep. 2020 Apr 6;10:5975. doi: 10.1038/s41598-020-62118-4 (PMC7136265; doi:10.1038/s41598-020-62118-4)
Supplement: Supplementary file 1 — Appendices A-E. [file 41598_2020_62118_MOESM1_ESM.pdf]

Models with environmental drivers offer a plausible mechanism for the rapid spread of infectious disease outbreaks in marine organisms

by EA Aalto[1], KD Lafferty[2], SH Sokolow[1], RE Grewelle[1], T Ben-Horin[3], CA Boch[4], PT Raimondi[5], SJ Bograd[6], EL Hazen[6], MG Jacox[6], F Micheli[1,7], and GA De Leo[1]

[1]*Hopkins Marine Station, Stanford University, Pacific Grove, CA, USA;*

[2]*United States Geological Survey, Western Ecological Research Center, at Marine Science Institute, University of California, Santa Barbara, CA, USA;*

[3]*Haskins Shellfish Research Laboratory, Rutgers University, Port Norris, NJ, USA;*

[4]*Monterey Bay Aquarium Research Institute, Moss Landing, CA, USA;*

[5]*University of California, Santa Cruz, CA, USA;*

[6]*NOAA Southwest Fisheries Science Center, Monterey, CA, USA;*

[7]*Stanford Center for Ocean Solutions, Pacific Grove, CA, USA*

Appendix A: Linearization of coastline using SSWD data

To linearize the Pacific coastline, we fit two linear regressions and calculated distance along the linear projection by mapping each point to the closest point on the line, then converting line distance from degrees latitude and longitude to kilometers. Because the width of a degree longitude varies substantially with latitude, we used separate values for each line calculated by averaging from the start and end points. This linearization is intended to facilitate a simple and purely qualitative comparison of SSWD observations with our model results, and does not incorporate the actual coastal geography, oceanographic conditions relevant to dispersal distance, or complex features such as Puget Sound. The same linearization was used to create averaged SST and along-shore current velocity values from the ROMS data.

Appendix B: Population and disease dynamics

EnvDr-Q-SEI *model:*

The class dynamics for the *EnvDr*-Q-SEI epidemiological model are described by the following set of discrete-time equations:

$$S_{i,t+1} = \sigma_S S_{i,t} e^{-\beta Q_{i,t}} + R(N_{i,t}) \quad \text{(B.1a)}$$

$$E_{i,t+1} = \sigma_E E_{i,t}(1 - \zeta(DD_{i,t})) + \sigma_S^{\tau} S_{i,t-\tau}(1 - e^{-\beta Q_{i,t-\tau}}) \quad \text{(B.1b)}$$

$$I_{i,t+1} = \sigma_I I_{i,t} + \zeta(DD_{i,t})\sigma_E E_{i,t} \quad \text{(B.1c)}$$

where σ_x is daily survival proportion of class x (assumed to not vary with temperature), τ is the delay between initial exposure and membership in the E class (defined as an individual with an established but asymptomatic infection releasing propagules), β indicates pathogen-susceptible infection rate, and $\zeta(DD_{i,t})$ indicates transition proportion from E to I (fully symptomatic infection) based on background transition and/or cumulative degree-days within the cell (with the complement, 1- $\zeta(DD_{i,t})$, representing the proportion of E individuals that remain asymptomatic). $R(N_{i,t})$ is a recruitment term limited by local population:

$$R(N_{i,t}) = \begin{cases} 0 & if\ N_{i,t} > K_i \\ K_i - N_{i,t} & if\ K_i - N_{i,t} < R_i \\ R_i & otherwise \end{cases} \quad \text{(B.2a)}$$

$$N_{i,t} = S_{i,t} + E_{i,t} + I_{i,t} \quad \text{(B.2b)}$$

where K_i is carrying capacity within the cell and R_i is a constant daily recruitment. Both cell-specific values are set to default values (K and R), then scaled by region (Table C2).

$Q_{i,t}$ indicates pathogen input into cell i:

$$Q_{i,t} = \sum_{j=1}^{C} \rho(j,i,t)(\varphi_E(E_{j,t} + \gamma I_{j,t}) + \sigma_Q Q_{j,t-1}) \qquad \text{(B.3)}$$

where C is the number of cells, φ_E is the per-capita pathogen production for class E, γ is a scaling constant for the increased propagule production of class I, and σ_Q is the persistence parameter for propagules within the cell. The proportion arriving from a particular cell j into cell i is computed as:

$$\rho(j,i,t) = \int_{(j-i-\frac{1}{2})}^{(j-i+\frac{1}{2})} \frac{1}{\sqrt{2\pi\theta_Q^2}} e^{\frac{(z*c_W+v_{j,t})^2}{2\theta_Q^2}} dz \qquad \text{(B.4)}$$

with $v_{j,t}$ indicating mean along-shore current near cell j at time t, cell width c_w, and standard deviation θ_Q. The dispersal kernel used here combines a long-range asymmetric transport process (the along-shore current) with a short-range Gaussian diffusion-advection process (the specific destination cell post-transport). Note that Eq. B3 evaluates all possible source cells for a destination cell and thus Eq. B4 inverts the typical dispersal calculation. To simplify, we assume that the propagule source is a single point at the midpoint of the source cell and that the current at the source cell represents conditions for the entire time step. We then integrate across the destination cell width to convert distance into cell-to-cell transport. Our model makes the assumption that a virion – or other infectious agent – is not absorbed after it contacts a host (see below for implications).

Transition from E to I occurs within a cell at a constant proportion p_{BG} (=0 for the purely environmentally-driven model) or when cumulative degree-days $DD_{i,t}$ approach or exceed a threshold:

$$\zeta(DD_{i,t}) = \begin{cases} 1 & if\ DD_{i,t} \geq DD_{thresh} \\ \max\left[p_{BG}, \left(\frac{DD_{i,t}}{DD_{thresh}}\right)^c\right] & otherwise \end{cases}. \quad \text{(B.5)}$$

The scaling exponent c governs the degree to which partial transition occurs as stress levels approach the threshold. Degree-days accumulate exponentially above a local threshold for extreme temperatures:

$$DD_{i,t} = DD_{i,t-1} + \begin{cases} a{A_{i,t}}^b & for\ T_{i,t} \geq T_{min} \\ -DD_{recover} & for\ T_{i,t} < T_{min}\ and\ DD_{i,t-1} \geq DD_{recover} \\ -DD_{i,t-1} & for\ T_{i,t} < T_{min}\ and\ DD_{i,t-1} < DD_{recover} \end{cases} \quad \text{(B.6)}$$

where a and b are scaling parameters, $A_{i,t}$ indicates temperature anomaly in cell i at time t, T_{min} indicates the minimum absolute temperature for temperature anomalies to be considered stressful, and $DD_{recover}$ indicates the maximum amount of accumulated stress recovered on non-anomalous days. Alternative forms of the stress function are detailed in Appendix D.

Both $T_{i,t}$ and $A_{i,t}$, the daily mean sea surface temperature and temperature anomaly in cell i at time t, are calculated using ROMS data for the Pacific coast from January 1st, 2013 through December 31st, 2015[49]. Anomaly is defined as deviation from local mean seasonal cycle as determined using 1999-2011 climate data. We linearized the data as described in Appendix A and averaged across gaps to match the spatial layout of the model (2500 km coastline in 5 km

cells). We used the same approach to transform the daily along-shore surface current $v_{i,t}$ from the ROMS model.

Const-Q-SEI *model*

The *Const*-Q-SEI model is a special case of the *EnvDr*-Q-SEI model for which p_{BG}=1.0 and τ=20. Consequently, exposed *S* class individuals develop a full infection after 20 days, transforming to *E* class, then transition to *I* after a single timestep.

EnvInf-Q-SEI *model*

The *EnvInf*-Q-SEI model represents an extension of *Const*-Q-SEI for which temperature affects the speed of disease dynamics, but does not trigger transition from *E* to *I*. The first temperature effect is through the strength of the disease transmission term β (see Eqs. B1a & B1b). In Ben-Horin et al. [45], abalone withering syndrome prevalence was found to increase with daily temperature fluctuation. Inspired by this, in the *EnvInf*-Q-SEI model the rate of susceptible infection is not constant but assumed to vary linearly with scaled temperature variance:

$$\beta(\Delta T_{i,t}) = \beta \Delta T_{i,t} \tag{B.7}$$

where $\Delta T_{i,t}$ is a 5-time-step moving variance average scaled such that the mean daily value across the simulation =0.5, or 50% of the transmission rate for *Const*-Q-SEI, but ranges from close to 0 on cold days to 10x or greater on the hottest days.

Secondly, disease progression is accelerated during periods of high temperature[39,41]. For *Const*-Q-SEI, there is a constant delay transitioning from *S* to *E* which, for the default value of

τ=20, always takes 20 timesteps to complete. For *EnvInf*-Q-SEI, however, higher temperatures speed up this transition: disease progression doubles for every 3 °C above the T_{cold} threshold, to a maximum of 4x faster at 6+ °C. Thus, a single high temperature day advances the disease 2-4x as fast as a cold day, and the complete *S->E* transition could take anywhere from 5-20 timesteps depending on the proportion of hot and cold days in the incubation period following initial infection.

Virion loss

Virion loss upon sea star contact is likely a relatively small source of pathogen loss compared to other sources. Furthermore, because each cell in our model has similar host density, there would be no variation in relative loss rate due to this mechanism. If we were modeling filter feeders, loss due to host contact might be quite high, causing us to over-estimate infection rates. This assumption would have been most problematic for dose-dependent exposure in a filter feeder with variable density[66]. In such a case, the assumed loss of virions upon infection in dense host populations could keep virion densities below the infective dose, preventing disease spread. Such inverse density dependence could set up spatially patchy mass mortalities that impact low density host populations, but this scenario does not appear to fit SSWD.

Appendix C: Model parameters

Table C1: Model parameters

Parameters	Symbol	Value	Range tested[a]	
Demographic				
Daily survival of *S*	σ_S	0.99992	+/- 25% of rate[b]	c
Daily survival of *E*	σ_E	0.99992	+/- 25% of rate[b]	c
Daily survival of *I*	σ_I	0.135	+/- 25% of rate[b]	d
Daily recruitment	R	0.2 ind km^{-1} day^{-1}	+/- 25%	e
Default within-cell carrying capacity	K	10^3 ind km^{-1}	-	f
Disease				
Infection parameter	β	10^{-1} $prop.^{-1}$	10^{-4} - 10^1	g
Delay between exposure and full infection	τ	20 days	5 - 25	h
Pathogen propagule production for *E*	φ_E	0.1 prop. ind^{-1}	+/- 25%	g
Scaling constant for class *I* production	γ	50	10 - 100	g
Pathogen propagule persistence	σ_Q	$(0.075)^4$	0.005^4 – 0.95^4	i
Constant *E*-to-*I* transition proportion	p_{BG}	0.0-1.0	-	
Spatial and environmental				
Length of coastline	C	100 cells	-	
Cell size	c_w	5 km	-	
Propagule dispersal standard deviation	θ_Q	5 km	+/- 25%	
Cumulative degree-day threshold	DD_{thresh}	500 °C	-	j
Degree-day linear scaling parameter	a	1.0	-	j
Degree-day exponential scaling parameter	b	1.5	0 - 2	j
Degree-day recovery value	$DD_{recovery}$	5 °C	0 - 10	k
Degree-day partial transition exponent	c	3	-	l
Minimum stress temperature	T_{min}	12 °C	-	m
Maximum infection delay temperature	T_{cold}	9 °C	-	m

a. The results of the range sensitivity tests are given in Appendix D.
b. The +/- sensitivity range was applied to the equivalent mortality rate, rather than the survival parameter itself.
c. Daily survival of the S and E classes is based on an estimated 34.1 year lifespan from Menge 1975.
d. Daily survival of the I class is based on an average time until death of 12 hours, in keeping with the rapid decline observed in the field.

e. Daily recruitment of mature individuals is approximated using the 0.0152 ind/m^2 estimate from Menge 1975 and the total habitat per cell (see below), scaled using data from Miner et al. 2018.
f. Default carrying capacity was set to 10,000, equivalent to the 0.52 ind/m^2 estimate from Menge 1975 multiplied by a 5,000 m coastline per cell and approximately 3.85 m of available vertical habitat. This was then scaled per-cell by the relative densities seen in Miner et al. 2018.
g. The infection parameter was chosen by comparing MLE values across several orders of magnitude and choosing the best compromise value between density and prevalence likelihoods across the three models.
h. Maximum delay roughly equal to mean 9 °C mortality time in Kohl et al. 2016.
i. Pathogen propagule persistence was set to 7.5% survival after each 6-hour period (Hewson et al. 2018), raised to the 4th power to approximate daily persistence. We tested a broad range of possible 6-hour persistence values.
j. The various stress accumulation parameters were set by comparing MLE values across several different possible accumulation functions and choosing the values which produced the best compromise between density and prevalence likelihood (see Appendix D).
k. The per-day stress recovery during non-anomalous conditions was set to be approximately equal to the stress from a moderate 3 °C anomaly.
l. We added partial transition of some individuals as the stress threshold was approached to avoid an artifical knife-edge transition threshold. We chose a simple scaling exponent to allow approximately 20% of individuals transition at about 75% of the stress threshold, but less than 2% at 50%.
m. Chosen as the cold-water and warm-water experimental temperatures in Kohl et al. 2016.

Table C2: Regional density and recruitment (approximated from Miner et al. 2018)

Region	Start of region[a]	Relative initial density (2013)[b]	Relative recruitment[b]	2014 depletion	2015 depletion
CA - South	0 km	0.25	1x	99%	99%
CA - Central	750 km	0.5	20x	75%	75%
CA - North	1200 km	0.5	50x	30%	75%
OR	1750 km	1.0	50x	30%	75%
WA	2150 km	1.0	20x	30%	75%

a. Southern boundary of the region as measured in km from the southern boundary of our simulated coastline. Approximated to fit cell divisions.
b. Density and recruitment levels relative to the other regions.

Appendix D: Sensitivity to stress, outbreak, and model parameters

Note: in all referenced parameter sensitivity tables except Table D6, *italics* are used to indicate the results for the parameter default and **bold** is used to indicate the minimal MLE value across parameter values for each category. The three MLE categories are: *density*, the MLE for assessing relative decline; *prevalence*, the MLE for assessing disease presence/absence; and, for overall comparison purposes, *combined*, the sum of *density* and *prevalence* (see Appendix E for MLE calculation details).

Stress-accumulation parameters

In our model, we make two broad assumptions about how temperature-related stress accumulation affects sea stars. Firstly, as shown in Equation B.6, we assume that stress accumulates non-linearly using an exponential function (repeated here for convenience):

$$DD = aA_{i,t}{}^{b}\ for\ T_{i,t} > T_{min} \tag{D.1}$$

where *a* and *b* are scaling parameters. Although some studies of intertidal organisms have shown a non-linear response to temperature (e.g., oyster mortality[57] and mussel growth[58]), the precise form of the relationship is unclear and may differ for sea stars. Coral bleaching, for example, is often modeled with stress as constant above a threshold (i.e., a step-function). Consequently, we tested several different possible stress functions to assess model sensitivity to the mechanism of stress accumulation (Figure D1). We found that the overall goodness of fit for the *EnvDr*-Q-SEI was relatively unaffected by the shape of the stress function (Table D1).

Secondly, we assumed that accumulated stress only affected the transition from the asymptomatic *E* class to the symptomatic *I* class. In reality, stress is likely to increase mortality for the uninfected *S* class as well. To test the importance of this assumption, we altered the constant survival terms σ_S and σ_E as follows:

$$\sigma_{i,x,t} = \sigma_x - m \cdot DD_{i,t} \tag{D.2}$$

where *x* indicates class, *m* is a constant stress-related mortality modifier, and $DD_{i,t}$ is the accumulated degree-days within cell *i* at time *t*. By varying *m*, we showed that increasing stress-related mortality shifted the model with minimal MLE values from *EnvInf*-Q-SEI to *EnvDr*-Q-SEI and, at very high levels, to *Const*-Q-SEI (Table D2).

Parameter estimation

We used MLE values to select model parameters that were hard to estimate from field data or experimental work. We chose the infection parameter β (Table D3), infection delay τ (Table D4), and initial infection location (Table D5) based on which value best balanced likelihoods for the density and prevalence data. Note that relative model support, and thus our overall results, stays consistent across most parameter values. In Table D6 we compare model likelihoods at different data aggregation levels, and show that the models are closest at the medium resolution we chose. The *EnvInf*-Q-SEI model is favored in both categories at finer aggregation and the *EnvDr*-Q-SEI model has a better prevalence MLE value at coarser aggregation. Note that MLEs cannot be compared across different aggregation levels.

Hypercube sampling of model parameters

Because this is an exploratory rather than explanatory model, we did not use specific real-world disease parameters and were instead focused on a qualitative comparison between the output and the SSWD data. However, the results could still depend on the specific parameters chosen rather than the structure of the model. We estimated demographic parameters for *P. ochraceus* from Menge[67] and set epidemiological parameters from experimental results and by selecting parameters to minimize the MLE values (Tables D3-5). To assess sensitivity to changes in the demographic and epidemiological parameters of the core disease model, we ran a Latin hypercube sampling analysis[68], allowing parameters to freely vary by +/- 25% (or across a specified range; see Table C1). The results from this analysis show that, although variance is introduced and there is a wider spread for the possible MLE values (especially for the density MLE and *Const*-Q-SEI; Figure D2a), the relative support for each model variant remains consistent across almost all parameter combinations (Figure D2b) and our overall conclusions remain the same. Note the relative lack of points in the lower-left corner of Fig. D2b (the "negative/negative" region), which shows that there were very few instances for which *Const*-Q-SEI was better on both metrics than the more environmentally-dependent models (though the reverse is often true), or *EnvInf*-Q-SEI than *EnvDr*-Q-SEI. In most instances, there was a trade-off between density and prevalence likelihood.

We used partial rank correlation coefficient (PRCC) analysis to determine which parameters significantly affected the relative difference between the models (Table D7). For each model comparison, a positive PRCC value indicates a parameter which positively correlates with a better fit for the more environmentally-dependent choice and a negative value indicates the reverse. The most significant parameters are those associated with propagule production (φ_E and

γ) and survival (σ_Q), and stress recovery ($DD_{Recovery}$). For prevalence likelihood, increased propagule production favors *EnvInf*-Q-SEI over *Const*-Q-SEI but *Const*-Q-SEI and *EnvInf*-Q-SEI over *EnvDr*-Q-SEI. These patterns are partially reversed for density likelihood and are mostly the same for propagule survival. Increasing stress recovery favors *EnvDr*-Q-SEI for density likelihood, while the opposite is true for prevalence likelihood.

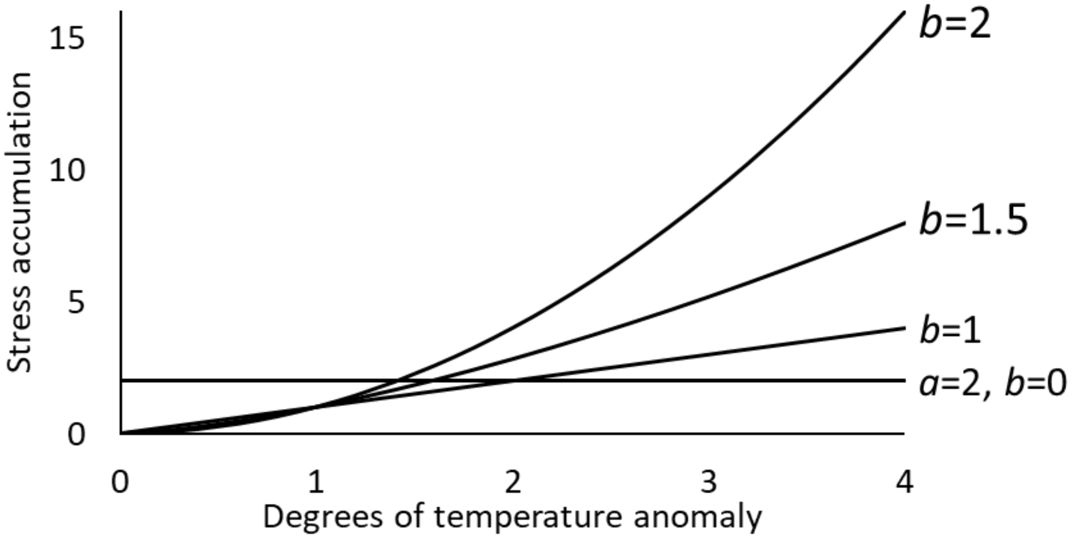

Figure D1. Stress-accumulation functions for different parameter combinations. The horizontal axis indicates the size of the temperature anomaly, and the vertical axis shows the accumulation of ‘degree-day’ stress. The lines indicate different combinations of scaling parameters, with *a*=1 unless otherwise indicated.

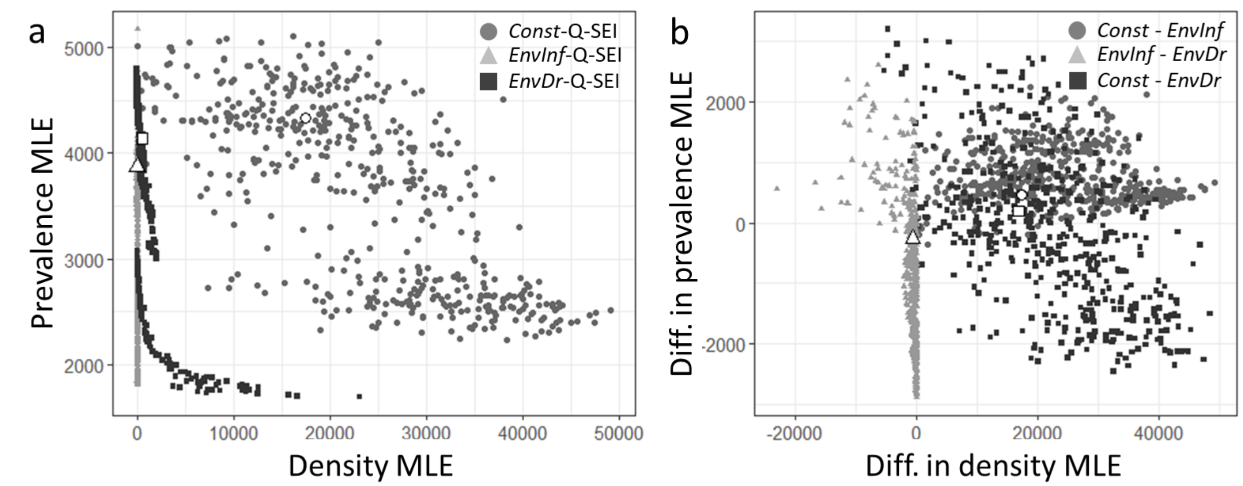

Figure D2. Absolute and relative MLE sensitivity to epidemiological parameters using hypercube sampling. For each of 500 sampling instances, ten parameters (recruitment, transmission strength, propagule dispersal, mortality rate of each stage, propagule production for infectious stages, and daily stress recovery) were independently sampled from uniform Latin hypercube distributions given in Table C1 (typically +/- 25% of default value). The default values for each scenario are shown by the larger, white shapes. a) The x-axis indicates the density MLE value for each model instance, and the y-axis indicates prevalence MLE values. b) The x-axis indicates the difference in density MLE values between the two models indicated, and the y-axis indicates the difference in prevalence MLE values. A positive value means that the first model was less supported by the data than the second model.

Table D1: MLE values for EnvDr -Q-SEI *with different stress accumulation parameters*

Stress func.	Density for DD_{thresh}=			Prevalence for DD_{thresh}=		
	500 °C	*250 °C*	*100 °C*	*500 °C*	*250 °C*	*100 °C*
b=2	**149**	153	69	**3868**	**3378**	**3272**
b=1.5	*586*	133	65	*4138*	3708	3416
b=1	521	**105**	**56**	4879	4167	3662
b=0, *a*=2	271	627	128	5065	4276	3515

Table D2: MLE values for varying levels of stress-related mortality on uninfected individuals

Window	Density for			Prevalence for		
	Const	*EnvInf*	*EnvDr*	*Const*	*EnvInf*	*EnvDr*
m=0	*17444*	***-0.05***	*586*	***4330***	***3873***	***4138***
m=0.005	300	71	**15**	4656	4080	4160
m=0.01	53	80	41	4778	4583	4191
m=0.02	**44**	94	64	5058	4774	4301
m=0.05	91	113	92	5204	5680	5914

Table D3: MLE values for different values of infection rate

β	Density for			Prevalence for		
	Const	*EnvInf*	*EnvDr*	*Const*	*EnvInf*	*EnvDr*
10	20793	**-69**	548	4346	2532	4145
1	16301	-59	546	**4317**	3479	4146
.1	*17444*	*-0.05*	*586*	*4330*	***3873***	*4138*
.01	**1631**	-23	**-12**	5107	6322	**4224**
.001	7898	16205	32	6233	7480	6326
.0001	8654	4461	2592	6963	6298	7335

Table D4: MLE values for different values of infection delay

Max delay	Density for		Prevalence for	
	Const	*EnvInf*	*Const*	*EnvInf*
5	35665	97	**1914**	**2056**
10	32933	60	3371	2528
15	27395	21	4578	3567
20	*17444*	*-0.05*	*4330*	*3873*
25	**13558**	**-23**	5189	4112

Table D5: MLE values for different initial infection locations

Initial cell	Density for		Prevalence for	
	Const	*EnvInf*	*Const*	*EnvInf*
125	**11767**	**-41**	4694	4340
250	*17444*	*-0.05*	*4330*	*3873*
375	26545	31	**3406**	**2257**

Table D6: MLE values for different aggregation window dimensions

Window	Density for			Prevalence for		
	Const	*EnvInf*	*EnvDr*	*Const*	*EnvInf*	*EnvDr*
25 km x 7 days	43080	**73**	821	4917	**4366**	4427
50 km x 14 days	*17444*	***-0.05***	*586*	*4330*	***3873***	*4138*
100 km x 30 days	9135	**15**	317	7070	6483	**5437**

Table D7: PRCC values and significance for MLE differences under hypercube sampling

Param	*Const - EnvInf*		*EnvInf - EnvDr*		*Const - EnvDr*	
	Density	Prev.	Density	Prev.	Density	Prev.
R	-0.001	-0.040	-0.023	0.032	-0.014	0.053
β	0.121**	0.054	0.108*	-0.079	-0.046	-0.096*
σ_S	0.022	0.088*	0.007	0.137**	-0.009	0.081
σ_E	-0.004	0.012	-0.007	0.052	-0.010	0.070
σ_I	0.133**	0.039	0.127**	-0.063	-0.067	-0.125**
σ_Q	0.731***	-0.356***	0.717***	-0.841***	0.125**	-0.857***
φ_E	0.186***	0.064	0.190***	-0.098*	0.035	-0.199***
γ	0.538***	0.373***	0.521***	-0.185***	-0.153***	-0.536***
θ_Q	0.027	0.050	0.013	0.152***	-0.029	0.105*
$DD_{Recovery}$	-	-	0.083	-0.863***	0.882***	-0.903***

*=p<0.05; **=p<0.01; ***=p<0.001

Appendix E: Maximum Likelihood Estimation

To form a likelihood-based approach to be maximized as model validation, we derive a likelihood function to describe the likelihood of each of the three models given the empirical data. This involves comparing model outputs to empirical findings matched both temporally and spatially. We define a likelihood function as:

$$L(\theta|x) = \sum_{i=1}^{N}\sum_{j=1}^{T}\log\left(p_{\theta_{ij}}(x_{ij})\right) \quad \text{(E.1)}$$

where θ denotes the model, x denotes empirical data, and i and j indicate cell and time period, respectively. We evaluated this likelihood function for both the density of sea stars and the prevalence of disease among the sea stars. Let ρ_{ij} be the observed density of sea stars in temporospatial window (i,j). Empirical and model density estimates approximate a lognormal distribution, so the likelihood function can be given as:

$$p_{\theta_{ij}}(x_{ij})_{dens} = \frac{1}{\rho_k(\log(1+\frac{\sigma_{ij}^2}{\mu_{ij}^2}))^{\frac{1}{2}}\sqrt{2\pi}} e^{\frac{-(\log(1+\rho_{ij})-\log\left(1+\mu_{ij}/\sqrt{1+\frac{\sigma_{ij}^2}{\mu_{ij}^2}}\right))^2}{2(\log\left(1+\frac{\sigma_{ij}^2}{\mu_{ij}^2}\right))}} \quad \text{(E.2)}$$

Because empirical density estimates occur on a yearly basis, the temporal window is a year in length. The model is evaluated in two-week increments, and μ_{ij} represents the mean of the 26 model density estimates for each year. Likewise, σ_{ij} is the standard deviation of the 26 model density estimates in each yearly window. This likelihood function evaluates relative decline by scaling the initial model density to match that of empirical density estimates in 2013.

SSWD prevalence in each temporospatial window is given by the model as π_{ij}. Each window is also assigned a 0 or 1 for empirical prevalence δ_{ij}, indicating whether SSWD was identified in that window. The number of surveys taken in each window can be denoted n_{ij} and the area surveyed per survey as α. The expression used to calculate the likelihood function for SSWD prevalence is as follows:

$$p_{\theta_{ij}}(x_{ij})_{prev} = \begin{cases} 1-(1-\pi_{ij})^{\alpha n_{ij}\lambda_{ij}}, & if\ \delta_{ij}=1 \\ (1-\pi_{ij})^{\alpha n_{ij}\lambda_{ij}}, & if\ \delta_{ij}=0 \end{cases} \quad \text{(E.6)}$$

Maximizing the resulting likelihood functions for density and prevalence, or minimizing -$L(\theta|x)$, gives the best fitting model to the empirical data.
